# Characterizing human movement patterns using GPS data loggers in an area of persistent malaria in Zimbabwe along the Mozambique border

**DOI:** 10.1186/s12879-022-07903-4

**Published:** 2022-12-15

**Authors:** Marisa Hast, Sungano Mharakurwa, Timothy M. Shields, Jailos Lubinda, Kelly Searle, Lovemore Gwanzura, Shungu Munyati, William J. Moss

**Affiliations:** 1 Johns Hopkins Bloomberg School of Public Health, Baltimore, MD USA; 2 Biomedical Research and Training Institute, Harare, Zimbabwe; 3 Africa University, Old Mutare, Mutare, Zimbabwe; 4 Telethon Kids Institute, Malaria Atlas Project, Nedlands, WA Australia; 5 School of Public Health, University of Minnesota, Minneapolis, MN USA

**Keywords:** GPS, Malaria, Zimbabwe, Population movement, Cross-border movement

## Abstract

**Background:**

Human mobility is a driver for the reemergence or resurgence of malaria and has been identified as a source of cross-border transmission. However, movement patterns are difficult to measure in rural areas where malaria risk is high. In countries with malaria elimination goals, it is essential to determine the role of mobility on malaria transmission to implement appropriate interventions.

**Methods:**

A study was conducted in Mutasa District, Zimbabwe, to investigate human movement patterns in an area of persistent transmission along the Mozambique border. Over 1 year, a convenience sample of 20 participants/month was recruited from active malaria surveillance cohorts to carry an IgotU^®^ GT-600 global positioning system (GPS) data logger during all daily activities. Consenting participants were tested for malaria at data logger distribution using rapid antigen diagnostic tests and completed a survey questionnaire. GPS data were analyzed using a trajectory analysis tool, and participant movement patterns were characterized throughout the study area and across the border into Mozambique using movement intensity maps, activity space plots, and statistical analyses.

**Results:**

From June 2016–May 2017, 184 participants provided movement tracks encompassing > 350,000 data points and nearly 8000 person-days. Malaria prevalence at logger distribution was 3.7%. Participants traveled a median of 2.8 km/day and spent a median of 4.6 h/day away from home. Movement was widespread within and outside the study area, with participants traveling up to 500 km from their homes. Indices of mobility were higher in the dry season than the rainy season (median km traveled/day = 3.5 vs. 2.2, P = 0.03), among male compared to female participants (median km traveled/day = 3.8 vs. 2.0, P = 0.0008), and among adults compared to adolescents (median total km traveled = 104.6 vs. 59.5, P = 0.05). Half of participants traveled outside the study area, and 30% traveled into Mozambique, including 15 who stayed in Mozambique overnight.

**Conclusions:**

Study participants in Mutasa District, Zimbabwe, were highly mobile throughout the year. Many participants traveled long distances from home, including overnight trips into Mozambique, with clear implications for malaria control. Interventions targeted at mobile populations and cross-border transmission may be effective in preventing malaria introductions in this region.

**Supplementary Information:**

The online version contains supplementary material available at 10.1186/s12879-022-07903-4.

## Background

Human mobility has been identified as an important driver for the reemergence or resurgence of malaria transmission in areas with previously successful malaria control [1–4]. Movement between high and low transmission settings can cause reimportation of malaria parasites if competent vectors are present, and cross-border movement in particular has been identified as a barrier to successful malaria control and elimination in areas with unstable transmission [5–7]. For these reasons, neglecting to account for human mobility patterns has been implicated in the failure of the Global Malaria Eradication Program [1, 8], and initiatives in recent years have been developed to address cross-border transmission as a barrier to malaria elimination in southern Africa [9–11]. To inform malaria control programs between countries and interrupt malaria transmission in border areas, it is essential to better characterize human mobility patterns in these regions.

Several methods of data collection have been utilized to investigate the impact of mobility on malaria transmission, including conducting travel surveys, investigating airline travel patterns and census migration records, and analyzing mobile phone data [6, 7, 12–17]. However, these approaches are limited by a lack of individual-level data due to aggregation to the population level to protect client privacy or aggregation to higher spatial levels such as cell towers. Individual-level data from surveys are additionally prone to recall biases and may not accurately measure the full extent of respondent movement [16, 18]. Furthermore, all methods relying on formal records have limitations in remote rural populations, which may have limited use of ticketed or documented travel mechanisms, and which may be more likely to participate in informal travel, including across borders.

Mobile devices that collect spatial information are an emerging method to investigate individual-level movement data at high spatial and temporal resolution. While cellular phones employed in population movement studies can transmit data in real time, their utility is limited in areas with developing infrastructure due to the potential scarcity of cell phone towers, unreliable power sources, and the high cost of data plans. However, portable global positioning system (GPS) data loggers have been found to successfully collect fine-scale individual-level movement data in remote areas with high levels of accuracy and high acceptability among participants [19–21]. These devices have been used to investigate the impact of movement patterns on transmission of various pathogens in resource-poor settings, including malaria, dengue, schistosomiasis, and hookworm [22–26].

To further characterize the relationship between human movement patterns and malaria transmission, we conducted a study in Mutasa District, Zimbabwe, using commercially available GPS data loggers. This region borders the Manica District of Mozambique to the east, and both formal and informal movement across the border is reported to be frequent for commerce, family and social networks, healthcare, and schooling, among other reasons [27–29]. The specific objectives of the investigation were to further describe human mobility in a remote region of southern Africa, to better understand patterns of cross-border movement, and to characterize the potential impact of these mobility patterns on malaria in an area of persistent transmission.

## Methods

### Study site

The study was conducted in the Honde Valley area of Mutasa District in eastern Zimbabwe between June 2016 and July 2017. The district encompasses approximately 622 km^2^ and is characterized by a wide range of elevation from 600 m in the eastern valleys along the Mozambique border to 2500 m in the mountains to the west. At the time of the study, the district had an estimated population of 174,000, most of whom were agricultural laborers [30]. Transmission of *Plasmodium falciparum* malaria in this region is highly seasonal, with little transmission occurring during the dry season from June to November and increased transmission during the rainy season from December to May [31, 32]. The predominant malaria vector is *Anopheles funestus*, and the primary malaria control interventions at the time of the study included mass distribution of insecticide-treated bed nets and indoor residual spraying (IRS) with the organophosphate insecticide pirimiphos-methyl (Actellic^®^ 300 CS, Syngenta, Sweden), in addition to other interventions such as intermittent preventive treatment of malaria in pregnancy [33]. Previous research in Mutasa District reported malaria prevalence to be approximately 6.4% and identified the area along the border with Mozambique as having an increased risk for malaria transmission, with risk declining with increasing distance from the border and higher elevation [31, 32].

### GPS data loggers

The data loggers used in the study were IgotU^®^ GT-600 devices (Mobile Action Technology, New Taipei City, Taiwan). These data loggers were selected based on their suitability for field use, including their light weight (37 g), large memory (> 250,000 points), long battery life, and accuracy in rural regions [19, 20, 26]. As previously described, these data loggers are reported to be accurate within 20 m at least 90% of the time under open sky and are reported to have an average error of 4.4 m while stationary and of 10.3 m while in motion [19, 20]. Data loggers were programmed to be motion-activated to preserve battery life, and the power button was disabled so that participants could not accidentally turn off the device. While in motion, loggers recorded latitude, longitude, date, and time every 5 min.

### Study population

The study population was recruited from two active surveillance cohorts of the Southern and Central Africa International Centers of Excellence for Malaria Research (ICEMR) [34], which has been operating in Mutasa District since October 2012. Household selection used a modified cluster sampling design. A 1 × 1 km grid was overlaid over Quickbird™ satellite images of the district (DigitalGlobe Services, Denver, CO), and grid quadrants were selected to be in either cross-sectional or longitudinal sampling cohorts using spatially balanced random sampling to ensure the inclusion of households across the region. In odd months, 25–30 new cross-sectional households were selected from the appropriate study grids using population proportional to size sampling and were visited only once. In even months, 25–30 randomly selected longitudinal households were visited six times per year. At the time of the study, most longitudinal households had been participating since the start of data collection in 2012, but households were occasionally replaced if they declined to participate further.

At each study visit, household coordinates were recorded, and all consenting household members provided a tympanic temperature and blood specimen by finger stick for hemoglobin testing and *P. falciparum* HRP-2 rapid diagnostic testing (RDT) (Standard Diagnostics, Kyonggi, Republic of Korea). A questionnaire was administered to participants 16 years or older and to guardians of children under 16 years, with questions on demographic characteristics, history of travel or healthcare seeking, and household malaria interventions, among other subjects. Participants with a positive RDT were offered treatment with Coartem^®^ (Novartis, Basel, Switzerland), the first-line standard of care in Zimbabwe. Additional study methods are described elsewhere [35–37].

### GPS data collection

Approval was received from local chiefs, and a pilot study was conducted in May 2016. Official recruitment began in June 2016 with the longitudinal cohort and continued every month until May 2017. For each month of data collection, a convenience sample of 20 participants from active malaria surveillance households was invited to carry a data logger for approximately 30 days. Any participant who declined was replaced with another participant to reach the goal of 20 per month. Replacement participants could be selected from the same household or from another household. Recruitment was restricted to participants aged 13 years or older, and no more than two members of the same household could be recruited during the same month. Data loggers could be worn around the wrist, around the neck, or in a pocket or bag, and participants were asked to carry the device at all times during normal daily activity except while sleeping, swimming, or bathing.

The evening prior to a scheduled ICEMR household visit, the study team visited selected households and informed participants about the option to participate in the GPS study in addition to the standard study activities. The study team explained the purpose of the GPS study, addressed concerns about confidentiality and distributed pamphlets translated into Shona, the local language. The following day at the study household visit, data loggers were distributed to consenting participants and all standard study procedures were conducted, including collection of blood specimens by finger stick and survey administration. Data logger serial numbers were matched to unique participant IDs, and the date and time of distribution were recorded. At logger collection approximately 30 days later, the full study visit procedures were repeated, including blood collection and the survey.

Cross sectional participants were recruited in odd months and longitudinal participants were recruited in even months. All longitudinal participants who successfully carried a logger in the first 6 months of the GPS study (June, August, or October 2016) were invited to participate a second time approximately 6 months from their original participation (December 2016, February, or April 2017). If an individual declined to repeat participation, they were replaced by another longitudinal study participant. Cross-sectional participants were not invited to participate for a second month. Therefore, approximately twice as many cross-sectional study participants were recruited to the GPS study as longitudinal study participants, but the overall number of logger distributions was the same between cohorts.

### Data management and processing

When GPS loggers were collected by the study team, they were transported back to the field office, and data were uploaded onto a password-protected computer using the @trip software specific to IgotU products. Data points not within the time interval of participation were removed, and data files were uploaded into REDCap secure file-sharing software [38]. Logged points and participant household locations were plotted in ArcGIS Version 10.7 (ESRI, Redlands, CA), and logger tracks for each participant were cleaned and mapped using a trajectory analysis tool developed for ArcGIS [26, 39]. For each logged point, distances to participant household, the study area boundaries, and the Zimbabwe border were calculated. Movement intensity plots were developed by season and participant characteristics using the ArcGIS trajectory analysis tool, which indicate the cumulative participant time spent in each location using a red to blue color scale. Images of logged data points and intensity plots were plotted over Digital Globe satellite images of the study area.

### Data analysis

Cleaned GPS data files were uploaded into STATA 15 (Stata-Corporation, College Station, TX) and merged by participant ID and date to household survey data for participant study visits conducted at logger distribution and collection. Participant malaria status by RDT, fever, and anemia was determined at each visit. Fever was considered tympanic temperature exceeding 38 °C, and anemia was defined by WHO criteria for hemoglobin levels by age and sex [40]. For each participant $i$, total participation time per logger track ${T}_{i}$ was calculated. The number of minutes spent at each logged point location ${\tau }_{ij}$ was estimated to be half the time between the previous and subsequent points. For each track, the proportion of time spent at the location of each logged point was calculated as the fraction of the time spent at that point over the total participation time for that track $({\tau }_{ij}/{\rm T}_{i})$. Participation time during peak mosquito biting hours ${\overline{\rm T} }_{i}$, was defined as all recorded time from 6 pm to 6 am [41–43], and the proportion of peak biting time at each location was calculated as ${(\tau }_{ij}/{\overline{\rm T} }_{i})$.

Participants were defined as being at home if they were within 50 m of their household, which was determined to be the distance that most accurately captured movement patterns in sensitivity analyses; however, it could not be determined whether participants were inside or directly outside their house due to limitations in the precision of the GPS logger devices. For each participant logger track, the proportion of time at home, total distance traveled, maximum distance from home, and average daily distance traveled were calculated for total time ${T}_{i}$ and for peak mosquito biting hours ${\overline{\rm T} }_{i}$. The median, interquartile range (IQR) and overall range from minimum to maximum value of these metrics were presented and compared by season (rainy vs. dry), age group (adults aged ≥ 18 years vs. adolescents aged 13–17 years), and sex (male vs. female) using Wilcoxon rank-sum tests. Travel out of the study area was determined using a shapefile developed for this study, and travel outside Zimbabwe into neighboring countries was determined using open-source Global Administrative Areas shapefiles [44].

### Activity space

To visualize participant activity space, plots were created of the cumulative proportion of time spent at increasing distances from the household, stratified by sex, age category, and season. Activity space plots were examined at the full spatial extent of distance traveled to compare patterns of large-scale movement (> 1 km). To examine fine-scale movement during peak mosquito biting hours, separate plots were developed within 1 km of the household.

### Ethical considerations

This study was approved by the Institutional Review Boards of the Johns Hopkins Bloomberg School of Public Health (IRB# 3467), the Biomedical Research and Training Institute (AP102/11), and the Medical Research Council of Zimbabwe (MRCZ/A/1625). The study team had previously sought permission from local chiefs to conduct the overall ICEMR study, and permission was obtained again for the nested GPS logger study. Informed consent was obtained from all participants or their legal guardian for the overall ICEMR study and also separately for the GPS logger study. Loggers were protected by a secure password, and data could only be downloaded using the associated @trip software and a unique connection cable, both of which were kept in a locked box in the secure field station. All methods were performed in accordance with the Declaration of Helsinki.

## Results

### Participant characteristics

Over 12 recruitment periods from June 2016 to May 2017, 240 data loggers were distributed to 191 participants from 122 unique households. Of these, 120 were recruited from cross-sectional households, and 71 were recruited from longitudinal households. Among the 60 longitudinal study participants recruited in the first six months of data collection (June, August, or October 2016), 49 (82%) agreed to carry the data logger again 6 months after their original month of participation. Eleven (18%) declined or were not available, and these individuals were replaced by a new longitudinal study participant.

The median participant age at logger distribution was 31 years (range 13–88 years), and 43% of participants were male (Table 1). Half of participants reported that their head of household was permanently employed, 45% reported using an open water source, and 16% lived in a household with a dirt floor. Fifty-two percent lived within 5 km of the Mozambique border, and 70% lived within 2.5 km of a health clinic, which was estimated to be approximately a 30-min walk. The altitude of households selected for participation ranged from 639 to 939 m above sea level, with approximately a quarter living above 800 m. Sixteen percent of participants reported living in a different home for part of the year, 17% reported visiting a health center in the previous 6 months, and 39% reported sleeping away from home at least one night in the previous month. Regarding malaria interventions, only 26% of participants reported sleeping under a bed net the previous night, but 89% reported a history of household IRS with pirimiphos-methyl.

**Table 1:** Participant characteristics at visit 1 (N = 191)

	n	%
Demographics		
Age < 18	28	14.7
Male	82	42.9
Household visit type		
Cross-sectional cohort	120	62.8
Longitudinal cohort	71	37.2
Household characteristics		
Head of household permanently employed	98	51.3
Household uses open water source	84	44.0
Household has dirt floor	31	16.2
Household above 800 m elevation	48	26.4
Within a 30-min walk^ of a health clinic	133	69.6
Within 5 km of Mozambique border	100	52.4
Reside at a different home part of the time	31	16.2
Health-related behaviors		
Sleeps under a bed net	49	25.7
House ever sprayed with pirimiphos-methyl	167	87.4
Visited health center in past 6 months	32	16.8
Slept away from home in the past month	73	38.2
Clinical results		
RDT positive	7	3.7
Fever at visit	3	1.6
Report fever in past 2 weeks	26	13.6
Anemic at visit	53	27.8

^2.5 km

At the time of logger distribution, 53 participants (28%) were anemic, three (1.6%) had a fever, and another 26 (14%) reported having a fever in the past 2 weeks. Seven participants (3.7%) tested positive for malaria by RDT. Four of the RDT-positive results occurred in March 2017, with the remaining three occurring in November 2016 and January 2017. The median age of participants with a positive RDT was 16 years (range 13–55), and 4 of the 7 (57%) were female.

At logger collection, a repeat study visit with RDT testing was successfully conducted for 213 (89%) of all 240 logger distributions. At the second study visit, four participants tested positive for malaria by RDT. These RDT-positives included two incident cases among male adults (February and March 2017) and two persistent positives among a male adult and a female adolescent (April 2017), which may have been incident cases or persistent parasitemia if they did not take the Coartem as directed.

### Participant movement patterns

**Fig. 1:**
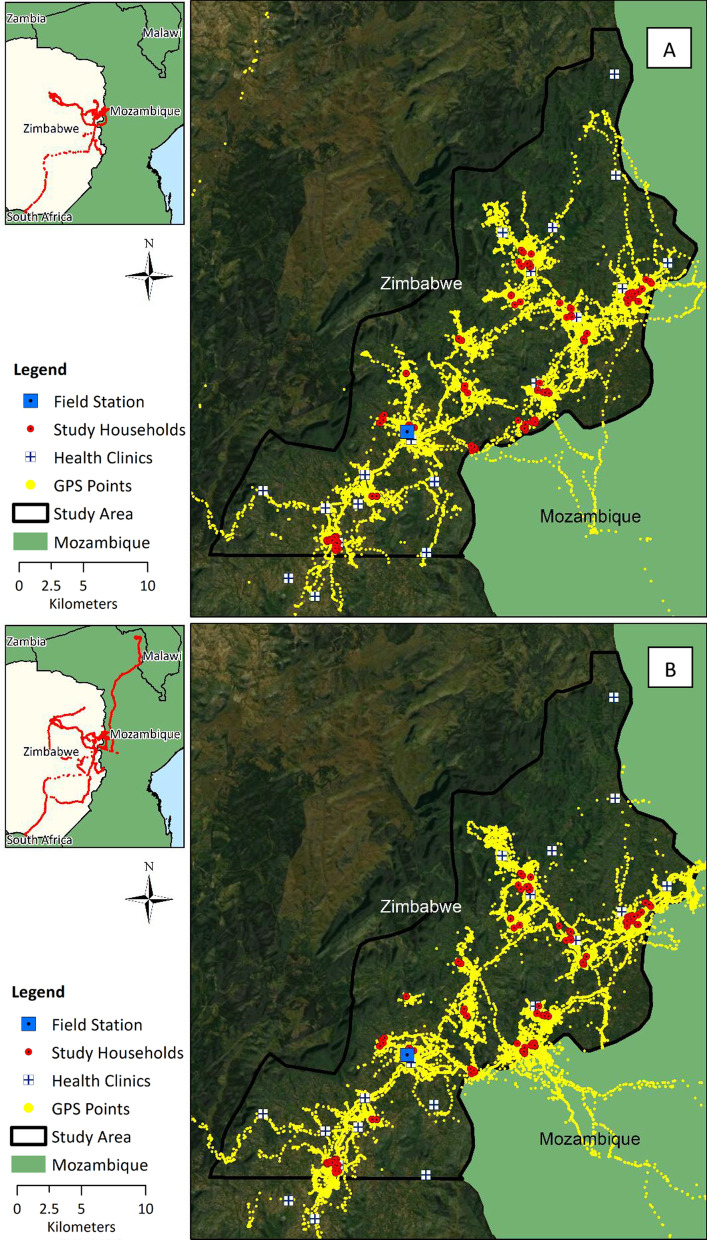
GPS data logger points stratified by **A** dry season and **B** rainy season. Longer distance travel is seen in insets in upper left of each panel

A total of 228 out of the 240 data loggers distributed yielded movement tracks that were usable for analysis (95%), and at least one usable movement track was collected from 184 of 191 participants (96%). Movement tracks were not considered usable if no points were recorded or if they had fewer than 100 points. Data loggers were collected from absent participants if the device was left at home or returned by another household member. Of the 184 participants who contributed movement tracks, 142 contributed 1 track, and 42 contributed 2 tracks approximately 6 months apart. The final cleaned dataset consisted of 356,707 data points, comprising 7771 person-days of data collection, and covering a combined distance of 49,999 km (Fig. 1). Over all collected points, there was an average of 6.4 km travelled per person-day of data collected.

**Fig. 2:**
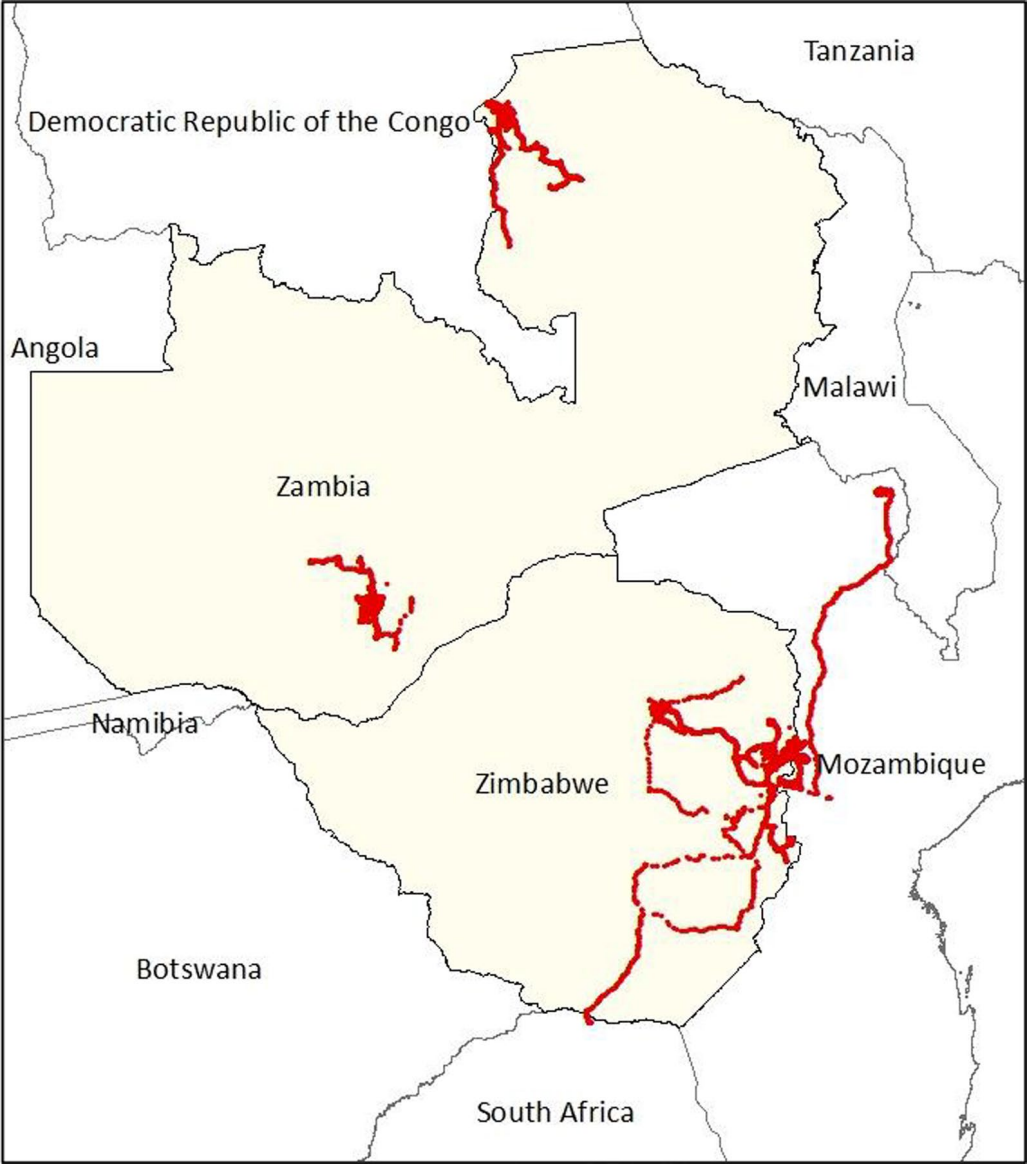
Range of participant movement in Mutasa District, Zimbabwe GPS data logger study compared to two GPS data logger studies in southern Zambia (Choma District) and northwest Zambia (Nchelenge District)

Across all movement tracks, there was a median of 5.4 min (IQR: 5.2–10.7) and a median of 15.5 m (IQR: 7.6–39.6) between recorded points. Based on the time and distance between points, only 0.3% of points were recorded at speeds greater than a typical human running pace (4.5 m/s), indicating that the participant may have been in a vehicle or on a bicycle. Per movement track, there was a median of 1,144 GPS points (IQR = 610–2,275; range = 115–9205) and 30.1 days of data (IQR = 29.0–39.0; range = 3.9–103.1). Also per movement track, total distance traveled per track ranged from 1.3 to 2360.3 km (median = 98.4; IQR = 32.9–228.8), average daily distance ranged from 0.04 to 90.5 km/day (median = 2.8; IQR = 1.1–7.5), and maximum distance from home ranged from 0.05 to 518.0 km (median = 5.0; IQR = 2.2–17.2). The movement range for this population compared to previous population movement studies in Zambia can be seen in Fig. 2.

Participants spent a median of 4.6 h away from home per day (IQR = 1.8–9.4; range = 0.01–23.9) and a median of 1.2 h away per night during peak biting hours (IQR = 0.4–3.4; range = 0.01–12). Seventy-nine participants (43%) spent at least 2 h away from home per night on average. Nineteen participants (10%) averaged > 20 h per day away from home per day during at least one movement track, indicating that they had a second household or were traveling during most of the data collection period. Seventy-nine participants (43%) spent time in Zimbabwe within 1 km of the Mozambique border, 103 (56%) spent time within 3 km, and 143 (78%) spent time within 5 km of the border. During peak biting hours, 56 participants (30%) spent time in Zimbabwe within 1 km of the border, 87 (47%) spent time within 3 km, and 115 (63%) spent time within 5 km.

Participant movement occurred broadly throughout the study area and ranged throughout eastern Zimbabwe and into Mozambique. By distance, 69 participants (38%) traveled more than 10 km away from their home, 31 (17%) traveled more than 50 km, 16 (9%) traveled more than 200 km, and 1 participant traveled more than 500 km. Ninety-four participants (51%) made at least one trip outside the study area, including 55 participants (30%) who traveled outside Zimbabwe. All participants who travelled outside the country crossed the border into Mozambique at least once. One of these also travelled to South Africa on two occasions, and another approached the border between Mozambique and Malawi but did not travel across.

The number of trips into Mozambique per movement track ranged from 1–50 (median = 2), and the amount of time spent in Mozambique per movement track ranged from 5 min to 37 days (median = 8.2 h). The individual who made 50 trips into Mozambique was an adolescent who lived very close to the border, and the majority of this travel is presumed to be informal. All other participants with > 10 trips into Mozambique were adults. Fifteen participants were logged to stay overnight in Mozambique for between 1 and 35 nights (median = 2). All participants who stayed overnight in Mozambique were adults, 53% were male, and 73% lived within 5 km of the border.

Among the 213 participant surveys conducted at logger collection, 48 (23%) participants reported sleeping away from home during their period of data logger collection, with trips lasting between 1 and 72 nights (median = 2.5). Six participants reported staying in another district in the province, seven reported staying in another province in Zimbabwe (6 of which stayed in Harare), and seven reported staying in Mozambique overnight. These reports do not match the GPS data exactly, likely due to recall biases or loss to follow-up. Among the 48 participants who reported overnight travel, 28 reported staying with family or friends, eight slept outdoors, and the rest slept in churches, schools, or guest lodges. Seventeen reported that the reason for travel was to visit family or friends, 11 attended funerals, and the rest traveled for work, commerce, healthcare, or other reasons.

### Seasonal movement patterns

**Fig. 3:**
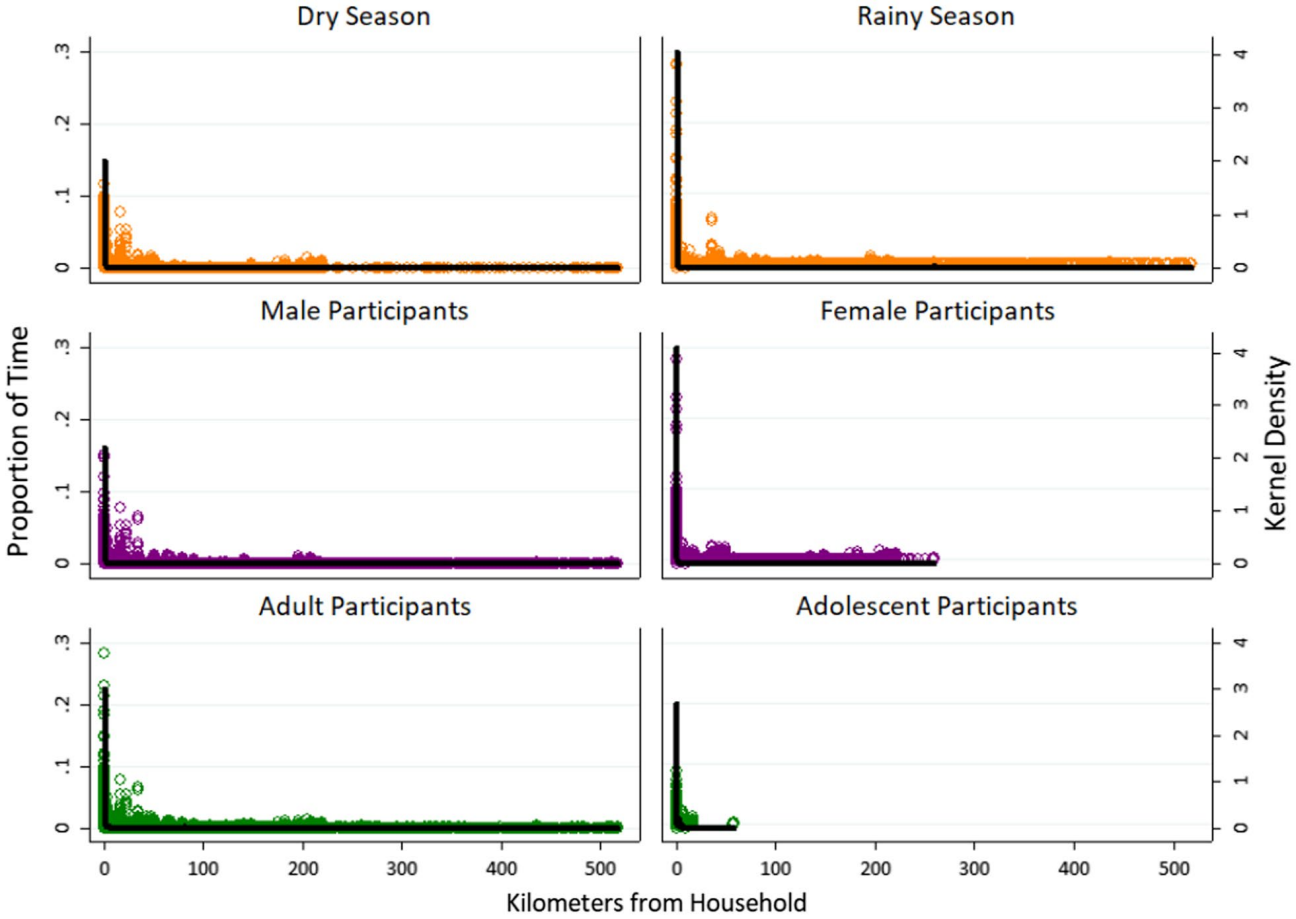
Activity space plots for participants showing proportion of time spent by distance from participant household, stratified by season of participation, sex, and age category

**Fig. 4:**
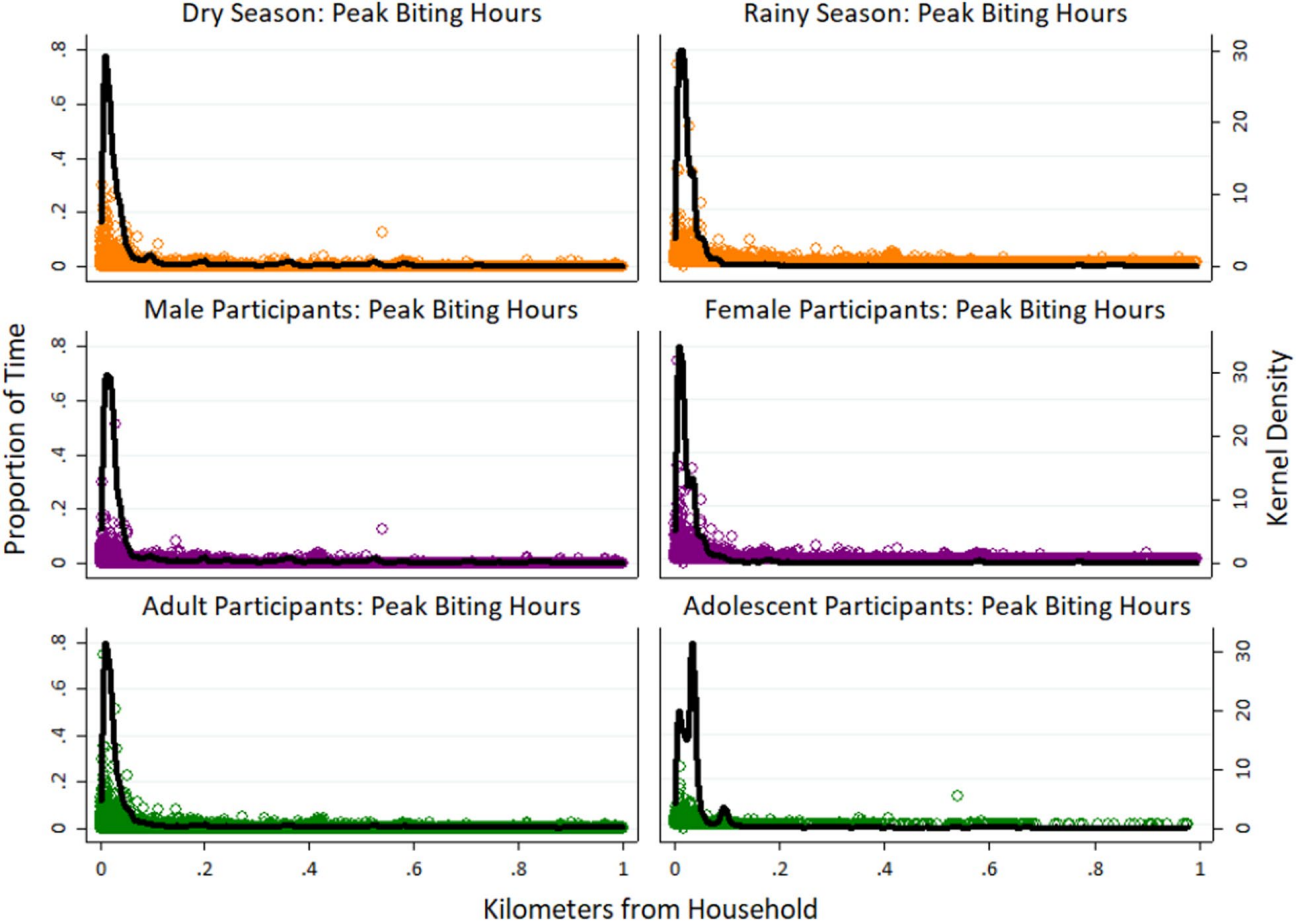
Activity space plots for participants showing proportion of peak vector biting time spent by distance from participant household, stratified by season of participation, sex, and age category

**Fig. 5:**
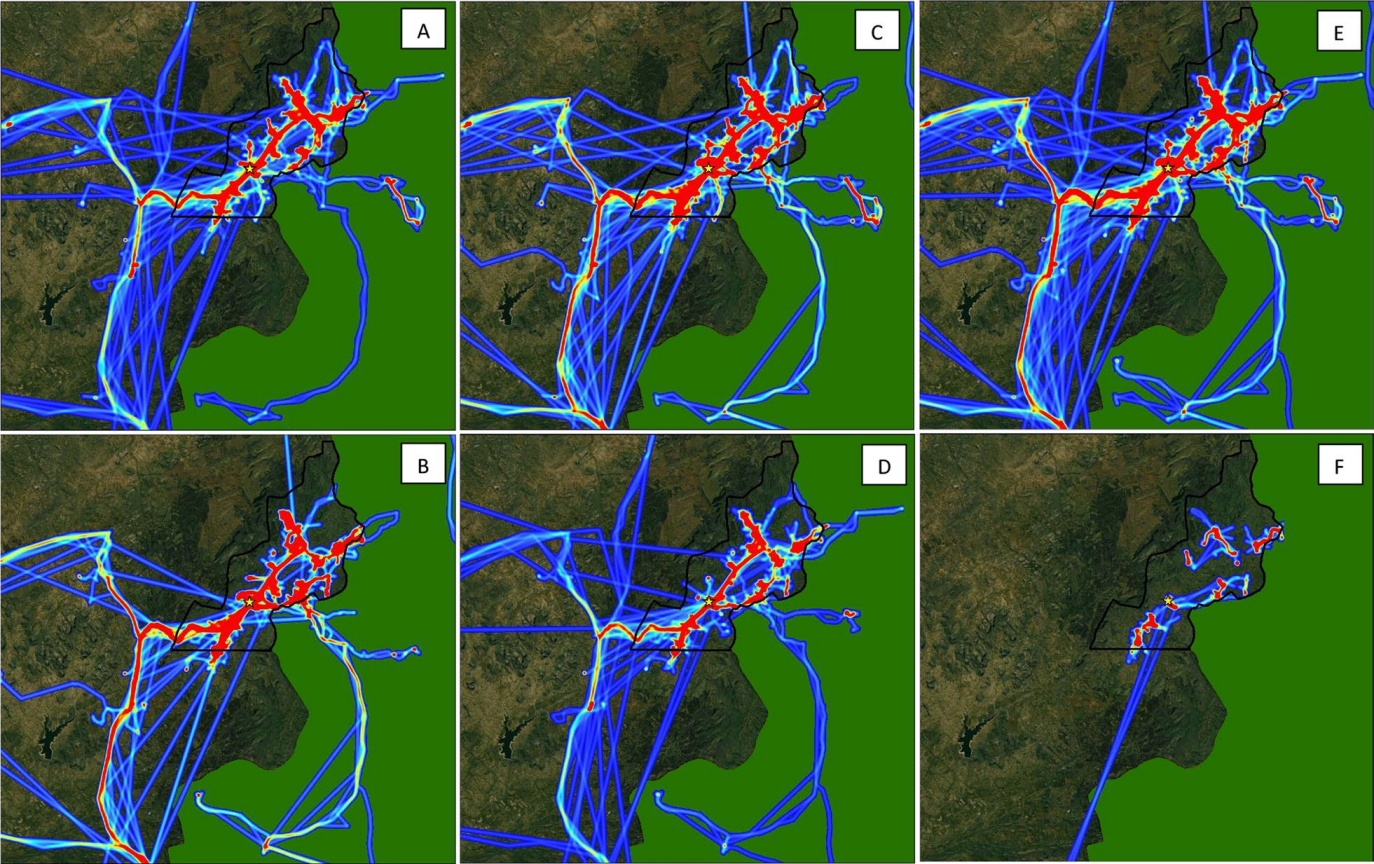
Intensity maps of population movement in Mutasa District from June 2016–May 2017 by season, gender, and age: **A** dry season [June, August, October], **B** rainy season [December, February, April], **C** male, **D** female, **E** adult [18+ years], **F** adolescent [13–17 years]. During periods of fast travel and/or periods where the logger was not recording points, intensity maps show straight line distances that may not reflect the true path traveled

**Fig. 6:**
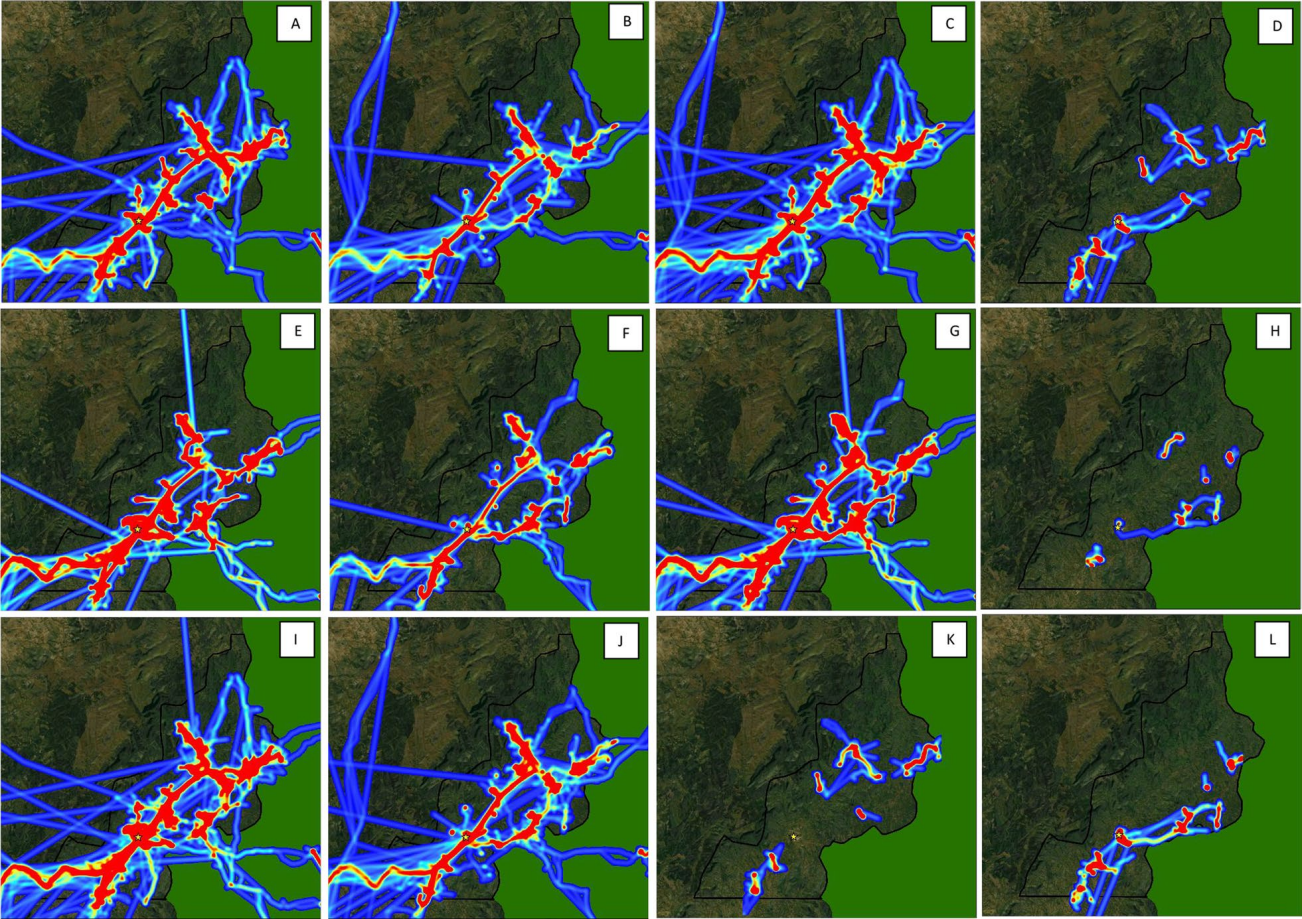
Intensity maps of population movement in Mutasa District from June 2016–May 2017 by: **A** Dry season, males, **B** dry season, females, **C** dry season, adults, **D** dry season, adolescents, **E** rainy season, males **F** rainy season, females **G** rainy season, adults **H** rainy season, adolescents **I** male adults **J** female adults **K** male adolescents **L** female adolescents. During periods of fast travel and/or periods where the logger was not recording points, intensity maps show straight line distances that may not reflect the true path traveled

Movement patterns in this region were somewhat seasonal, with higher metrics of mobility observed during the dry season (Table 2). These included higher average distance traveled per day, higher average number of hours away from home overall and per night, and higher maximum nighttime distance from home during the dry season. Total distance traveled per track did not vary by season, either overall or during peak mosquito biting hours. In activity space plots, participants also spent a higher proportion of time near their home during the rainy season (Fig. 3), although this difference was less evident at night (Fig. 4), and the maximum distance traveled from home was similar between seasons. Movement was widespread throughout the study area in both seasons, and both travel into Mozambique and long-distance travel occurred throughout the year (Fig. 1). However, in movement intensity maps disaggregated by season (Fig. 5A and B), travel into Mozambique appeared more frequent during the rainy season. In intensity maps disaggregated by demographics and season, travel was similar between rainy and dry seasons among adults and among participants of the same sex (Fig. 6); however, adolescents had higher mobility during the dry season compared to the rainy season (Fig. 6D and H). Intensity maps for each month of data collection can be found in Additional file 1: Figure S1. As a note, during periods of fast travel and/or periods where the logger was not recording points, intensity maps show straight-line distances that may not reflect the true path traveled. Examples of this could include if the logger lost the satellite signal when traveling through the mountains or was in a car trunk or glove compartment.

**Table 2:** Metrics of movement patterns among participants, stratified by sex, age, season, and peak vector biting hours

	Males		Females		P value*
	Median (IQR)	Range	Median (IQR)	Range	
Overall					
Total distance traveled (km)	140.3 (54.5–288.2)	1.3–2360.3	64.5 (27.9–181.7)	1.6–1260.9	*0.0008*
Average distance per day (km)	3.8 (1.6–9.6)	0.04–90.5	2.0 (0.8–6.0)	0.06–36.2	*0.0008*
Maximum distance from home (km)	5.8 (2.7–22.2)	0.05–518.0	4.7 (1.7–12.5)	0.05–260.1	0.1
Average hours away from home per day (> 50 m)	6.4 (2.7–10.3)	0.01–23.9	3.5 (1.2–8.1)	0.06–23.9	*0.006*
Peak mosquito biting hours^^^					
Total distance traveled (km)	19.9 (7.0–53.8)	0.4–1298.0	7.8 (2.5–21.4)	0.2–561.9	< *0.0001*
Average distance per night (km)	0.6 (0.2–2.0)	0.01–49.8	0.3 (0.1–0.7)	0.01–17.4	< *0.0001*
Maximum distance from home (km)	2.7 (1.0–18.7)	0.02–517.9	1.3 (0.2–9.9)	0.02–260.0	*0.005*
Average hours away from home per night (> 50 m)	1.6 (0.5–3.9)	0.01–12.0	0.9 (0.3–2.6)	0.01–12.0	*0.02*

	Adult (18+)		Adolescent (13–17)		P value*
	Median (IQR)	Range	Median (IQR)	Range	
Overall					
Total distance traveled (km)	104.6 (33.4–234.4)	1.5–2360.3	59.5 (29.6–121.5)	1.3–375.3	*0.05*
Average distance per day (km)	3.1 (1.1–7.6)	0.4–90.5	2.0 (1.1–3.4)	0.04–15.1	0.1
Maximum distance from home (km)	5.4 (2.4–21.1)	0.1–518.0	3.3 (1.8–5.1)	0.1–57.5	*0.02*
Average hours away from home per day (> 50 m)	5.1 (1.8–9.8)	0.01–23.9	3.7 (1.8–4.7)	0.03–23.8	0.1
Peak mosquito biting hours^^^					
Total distance traveled (km)	13.6 (4.2–39.4)	0.2–1298.0	6.6 (2.3–26.1)	0.3–71.5	*0.05*
Average distance per day (km)	0.4 (0.1–1.2)	0.01–49.8	0.2 (0.1–0.8)	0.01–2.3	0.1
Maximum distance from home (km)	2.3 (0.6–16.9)	0.02–517.9	1.1 (0.3–2.9)	0.03–16.8	*0.03*
Average hours away from home per night (> 50 m)	1.2 (0.4–3.7)	0.01–12.0	0.5 (0.3–1.7)	0.03–11.8	0.08

	Dry Season (June–November)		Rainy season (December–May)		P value*
	Median (IQR)	Range	Median (IQR)	Range	
Overall					
Total distance traveled (km)	116.7 (33.6–246.7)	1.6–1716.4	85.0 (29.1–219.9)	1.3–2360.3	0.1
Average distance per day (km)	3.5 (1.2–8.0)	0.1–52.2	2.2 (0.8–6.6)	0.04–90.5	*0.03*
Maximum distance from home (km)	5.8 (2.7–20.5)	0.1–517.9	4.3 (1.8–13.7)	0.1–518.0	0.07
Average hours away from home per day (> 50 m)	5.4 (2.2–10.6)	0.01–23.9	3.6 (1.2–7.9)	0.01–23.9	*0.02*
Peak mosquito biting hours^^^					
Total distance traveled (km)	15.7 (4.2–43.2)	0.2–1214.1	11.4 (3.4–32.8)	0.2–1298.0	0.2
Average distance per day (km)	0.5 (0.2–1.4)	0.01–33.6	0.3 (0.1–0.8)	0.01–49.8	0.06
Maximum distance from home (km)	2.8 (0.7–16.9)	0.03–517.1	1.5 (0.3–6.1)	0.02–517.9	*0.03*
Average hours away from home per night (> 50 m)	1.5 (0.5–4.0)	0.01–12.0	0.9 (0.3–2.7)	0.01–12.0	*0.05*

^*^Wilcoxon rank sum test; Wilcoxon rank sum *P* values are shown in italics if they are < 0.05

^Peak biting hours from 6 pm to 6 am

### Movement patterns by sex

Male participants had significantly higher mobility than female participants across almost all metrics. Male participants traveled a longer total distance per track, a longer average distance per day, and spent a higher average number of hours away from home (Table 2). These differences were also consistently observed during peak mosquito biting hours, with male participants traveling a longer maximum distance from home during these times. Similarly, in activity space plots (Fig. 3), male participants traveled a longer maximum distance from home, and female participants spent a higher proportion of time near the home overall and during peak biting hours (Fig. 4). In movement intensity maps, males had a higher footprint of travel throughout the study area and traveled more frequently into Mozambique (Fig. 5C and D). The higher mobility among male vs. female participants was also observed across seasons (Fig. 6), although the difference was particularly noticeable in the rainy season (Fig. 6E and F). Male adults had higher mobility across the study area (Fig. 6I and J), but male and female adolescents had similar movement patterns (Fig. 6K and L).

### Movement patterns by age

Similar to sex, adults had higher mobility than adolescents across most metrics. Adults traveled a longer total distance per track, traveled further from home, and spent more hours away from home compared to adolescents, both overall and during peak mosquito biting hours (Table 2). In activity space plots, adults traveled a longer maximum distance than adolescents (Fig. 3). However, on average, adults spent a higher proportion of time near their home both overall and during peak biting hours (Fig. 4). Intensity maps are challenging to interpret due to the much higher number of adult participants; however, few adolescents left the study area compared to adults, and only one traveled more than 10 km outside the study area (Fig. 5E and F).

Participant movement patterns did not vary significantly by longitudinal vs. cross-sectional cohort (Additional file 1: Figure S2) or by first vs. second movement track (Additional file 1: Table S1).

## Discussion

In this border region with persistent malaria transmission, participants consenting to carry a GPS data logger were highly mobile year-round within the study area, throughout Zimbabwe, and into neighboring countries. Although overall mobility was high, movement patterns were highly heterogeneous among individuals. A high proportion of participant time was spent near the home on average, and half of participants remained within 5 km of their household at all times; however, 30% of participants traveled out of Zimbabwe, 23% reported sleeping away from home, and nearly 10% traveled more than 200 km away. Movement indices were slightly higher during the dry season and were significantly higher among male participants and adults. Adolescents, in particular, tended to stay within the study area and remained near the home a higher proportion of the time. Thirty percent of participants travelled into Mozambique at least once, and 8% were recorded in Mozambique overnight with trips lasting up to 35 nights. Overall, these results agree with previous investigations of movement patterns in this region [45].

While 1-month malaria incidence in this population was low during the period of observation at only 1.9%, participant movement patterns have clear implications for malaria control. Over 40% of participants spent time within 1 km of the Mozambique border, which has been shown to have a higher risk of malaria transmission [31, 32]. During peak mosquito biting hours, 43% of participants spent at least 2 h away from home on average, during which time they may be more likely to be exposed to biting vectors. Furthermore, Manicaland Province, where Mutasa District is located, consistently has the highest number of malaria cases per year in Zimbabwe [46], and the high rate of participant travel throughout the country could further contribute to introductions in regions of lower relative transmission.

Perhaps most importantly, the high proportion of participants who traveled across the border into Mozambique may impact malaria control efforts in Zimbabwe. At the time of the study, Mozambique reported lower rates of key malaria control interventions and higher indices of transmission than Zimbabwe [45]. In particular, the Manica Province of Mozambique directly across the border from the study area has among the highest prevalence of malaria in the country, particularly in rural and border areas, with parasite prevalence among children reported to be 39% in the most recent Malaria Indicator Survey [47–49]. The frequency of cross-border travel, including overnight stays, thereby presents a clear risk of malaria importation from travelers into Mutasa District. This risk is further compounded by reported high rates of incoming cross-border movement from residents of Mozambique into Mutasa District to visit family, for healthcare seeking, for commerce, and other reasons [45]. Identifying individuals or groups at higher risk for malaria importation can aid in designing surveillance systems and malaria control interventions for these populations [6].

This investigation also further highlights the need for collaboration among neighboring countries for malaria control. Zimbabwe is one of the countries participating in the Elimination 8 Regional Initiative, aimed to accelerate malaria elimination in key countries in Southern Africa, and promotion of regional coordination among member countries and reduction of cross-border malaria are two of the key objectives [50]. Existing cross-border initiatives to reduce malaria transmission could serve as models for malaria control in this region [9–11, 51], and activities such as integrated surveillance systems, screening travelers at border crossings, and targeting economic migrants for interventions may reduce transmission across countries [5, 52, 53]. However, these interventions require further knowledge about formal and informal migration patterns, and efforts to understand the extent of formal and informal cross-border movement in particular settings are key to planning effective interventions in border populations.

To this end, the high heterogeneity of movement patterns among residents of Mutasa District in this study underscores the continuing need for multiple sources of individual-level data to fully capture the range of mobility behavior across different settings, accounting for local context and demographic factors. Of note, the population observed in this study traveled higher total distances, distances per day, and maximum distances from home compared to populations observed in Nchelenge District and Choma District in Zambia in similar studies (Fig. 2) [25, 26]. These individual and regional differences in small- and large-scale movement can be expected to influence malaria transmission risk [54–57]. Therefore, the suite of interventions needed for cross-border malaria control must vary according to local population movement patterns in addition to epidemiologic and environmental factors. This is particularly important as national malaria control programs move toward targeted interventions in areas of higher transmission [58–60].

This investigation was subject to several limitations. Participants were selected as a convenience sample within a larger surveillance study and are therefore not representative of the underlying population of Mutasa District. The exclusion of children from the sample in particular limits the ability to make inferences about the population most at risk of mortality and morbidity due to malaria. Furthermore, there were potential inaccuracies caused by limitations of the GPS logger devices. Although a 5-min interval provides a high degree of precision compared to most population movement studies, all movement between recorded points was not captured, and therefore the total distance traveled may be underestimated. Due to limits in the precision of individual points, participants could not be classified as indoors or immediately outside their home, so calculated time away from home may underestimate time spent outdoors. Compliance with study protocol was also difficult to assess with these devices since no biometric data was collected. Participants could have forgotten the logger at home, intentionally not worn it, or allowed it to be worn by another person. Since these scenarios were difficult to verify, logger data was analyzed as collected unless a specific issue was reported to the field team. A further potential discrepancy was the presence of two persistent positive RDT results at logger collection, which could indicate either new incident infection, persistent infection, or persistent HRP-2 antigen positivity following treatment. Several studies have reported false positive RDTs after a prior malaria infection due to HRP-2 antigen persistence, and therefore a 1-month interval may not be long enough to accurately detect incident infections with RDTs [61–64].

Despite these limitations, this study was able to collect a large quantity of high-quality movement data and captured significant migration patterns among participants in a remote rural area. The investigation built off previous population movement studies conducted in Peru and Zambia and was the first to capture significant cross-border movement, which has clear implications for malaria control in this population. Furthermore, the study had high acceptability in this population and data quality was high overall; 95% of loggers distributed yielded usable movement tracks, corresponding to 96% of participants who provided at least one usable data file. These results indicate that this technology continues to be a feasible and useful method for detailed population movement studies, including in remote areas.

## Conclusions

Study participants in Mutasa District, Zimbabwe, exhibited a high degree of mobility throughout the year compared to other similar populations in southern Africa. Participants traveled long distances from home, including significant cross-border movement into neighboring Mozambique. Due to high levels of malaria transmission in Manica Province in Mozambique, these mobility patterns may have significant implications for malaria elimination in this region. Collaborations between countries to interrupt malaria transmission and prevent cross-border introductions could be effective in this region. Human mobility patterns and cross-border movement in particular should be considered in similar settings when designing targeted strategies for malaria control.

## Supplementary Information

**Additional file 1: Table S1.** Metrics of movement patterns among participants, stratified by track number and peak vector biting hours. **Figure S1.** Intensity maps of population movement in Mutasa District from June 2016–May 2017 by month: (A) Jun 2016; (B) July 2016; (C) August 2016; (D) Septembers 2016; (E) October 2016; (F) November 2016; (G) December 2016; (H) January 2017; (I) February 2017; (J) March 2017; (K) April 2017; (L) May 2017. **Figure S2.** Intensity maps of population movement in Nchelenge District from June 2016–May 2017 by cohort and season: (A) longitudinal cohort, dry season [June, August, October], (B) cross-sectional cohort, dry season [July, September, November], (C) longitudinal cohort, rainy season [December, February, April], (D) cross-sectional cohort, rainy season [January, March, May].

## Data Availability

The datasets generated and/or analyzed in this study are not publicly available due to privacy concerns for participants but are available from the corresponding author on reasonable request.

## Abbreviations

GPS: Global positioning system
IRS: Indoor residual spraying
ICEMR: International Centers of Excellence in Malaria Research
RDT: Rapid diagnostic test
