# Supplementary material for: Characterizing human movement patterns using GPS data loggers in an area of persistent malaria in Zimbabwe along the Mozambique border
Source: BMC Infect Dis. 2022 Dec 15;22:942. doi: 10.1186/s12879-022-07903-4 (PMC9756631; doi:10.1186/s12879-022-07903-4)
Supplement: Supplementary file 1 — Additional file 1: Table S1. Metrics of movement patterns among participants, stratified by track number and peak vector biting hours. Figure S1. Intensity maps of population movement in Mutasa District from June 2016–May 2017 by month: (A) Jun 2016; (B) July 2016; (C) August 2016; (D) Septembers 2016; (E) October 2016; (F) November 2016; (G) December 2016; (H) January 2017; (I) February 2017; (J) March 2017; (K) April 2017; (L) May 2017. Figure S2. Intensity maps of population movement in Nchelenge District from June 2016–May 2017 by cohort and season: (A) longitudinal cohort, dry season [June, August, October], (B) cross-sectional cohort, dry season [July, September, November], (C) longitudinal cohort, rainy season [December, February, April], (D) cross-sectional cohort, rainy season [January, March, May]. [file 12879_2022_7903_MOESM1_ESM.docx]

Additional file

Table S1: Metrics of movement patterns among participants, stratified by track number and peak vector biting hours.

|  | | Track 1 | | Track 2+ | | | | |  |
| --- | --- | --- | --- | --- | --- | --- | --- | --- | --- |
|  | | Median (IQR) | Range | Median (IQR) | | Range | | | P value* |
| **OVERALL** | |  |  |  | |  | | |  |
| Total distance traveled (km) | | 98.5 (33.6 – 235.2) | 1.6 – 2,313.1 | 91.0 (13.1 – 219.9) | | 1.3 – 2,360.3 | | | 0.2 |
| Average distance per day (km) | | 2.9 (1.1 – 7.7) | 0.1 – 77.5 | 2.6 (0.6 – 6.5) | | 0.04 – 90.5 | | | 0.1 |
| Maximum distance from home (km) | | 5.2 (2.5 – 18.2) | 0.1 – 518.0 | 3.4 (1.3 – 16.4) | | 0.1 – 518.0 | | | 0.08 |
| Average hours away from home per day (>50m) | | 4.9 (2.1 – 9.9) | 0.01 – 23.9 | 3.1 (1.0 – 7.0) | | 0.01 – 23.8 | | | *0.02* |
|  | |  |  |  | |  | | |  |
| **PEAK MOSQUITO BITING HOURS**^^^ | |  |  |  | |  | | |  |
| Total distance traveled | | 12.4 (4.1 – 38.2) | 0.2 – 1,214.1 | 13.4 (2.8 – 35.2) | | 0.4 – 1,298.0 | | | 0.6 |
| Average distance per day | | 0.4 (0.1 – 1.2) | 0.01 – 33.6 | 0.4 (0.1 – 0.9) | | 0.01 – 49.8 | | | 0.6 |
| Maximum distance from home | | 2.5 (0.6 – 13.1) | 0.02 – 517.1 | 1.1 (0.3 – 3.9) | | 0.02 – 517.9 | | | 0.07 |
| Average hours away from home per night (>50m) | | 1.4 (0.4 – 3.7) | 0.01 – 12.0 | 0.9 (0.4 – 1.6) | | 0.06 – 12.0 | | | 0.1 |
|  |  | |  | |  | |  |  | |
|  | | Longitudinal Cohort | | Cross-sectional Cohort | | | | |  |
|  | | Median (IQR) | Range | Median (IQR) | | Range | | | P value* |
| **OVERALL** | |  |  |  | |  | | |  |
| Total distance traveled (km) | | 99.8 (31.4 – 221.7) | 1.3 – 2,360.4 | 92.1 (33.8 – 251.3) | | 3.6 – 2,313.1 | | | 0.7 |
| Average distance per day (km) | | 2.8 (0.8 – 6.6) | 0.04 – 90.5 | 2.9 (1.1 – 8.0) | | 0.1 – 77.5 | | | 0.4 |
| Maximum distance from home (km) | | 4.8 (1.9 – 14.3) | 0.05 – 518.0 | 5.1 (2.4 – 20.3) | | 0.09 – 443.7 | | | 0.2 |
| Average hours away from home per day (>50m) | | 3.7 (1.2 – 9.5) | 0.01 – 24.0 | 5.3 (2.2 – 9.3) | | 0.06 – 24.0 | | | 0.1 |
|  | |  |  |  | |  | | |  |
| **PEAK MOSQUITO BITING HOURS**^^^ | |  |  |  | |  | | |  |
| Total distance traveled | | 14.8 (3.3 – 36.6) | 0.2 – 1,298.0 | 12.0 (4.2 – 39.4) | | 0.2 – 990.9 | | | 0.9 |
| Average distance per day | | 0.4 (0.1 – 1.0) | 0.01 – 49.8 | 0.4 (0.1 – 1.4) | | 0.01 – 33.2 | | | 0.7 |
| Maximum distance from home | | 1.8 (0.4 – 6.8) | 0.02 – 517.9 | 2.6 (0.05 – 16.8) | | 0.02 – 438.2 | | | 0.2 |
| Average hours away from home per night (>50m) | | 0.9 (0.3 – 2.6) | 0.02 – 12.0 | 1.6 (0.04 – 4.0) | | 0.01 – 12.0 | | | 0.06 |
|  | |  |  |  | |  | | |  |

*Wilcoxon rank sum test

^Peak biting hours from 6 pm to 6 am

Figure S1: Intensity maps of population movement in Mutasa District from June 2016 – May 2017 by month: A) Jun 2016; B) July 2016; C) August 2016; D) Septembers 2016; E) October 2016; F) November 2016; G) December 2016; H) January 2017; I) February 2017; J) March 2017; K) April 2017; L) May 2017

D

C

B

A


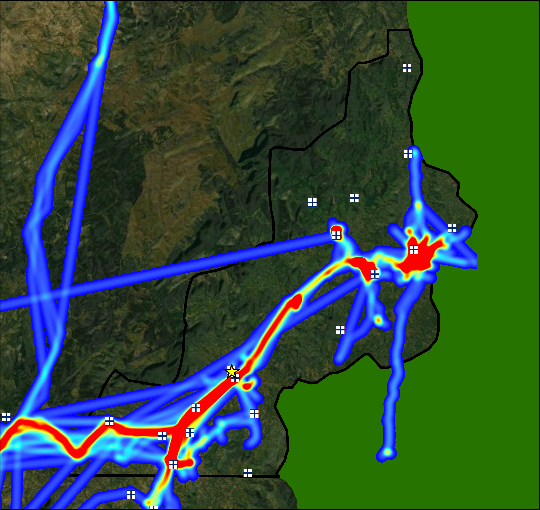

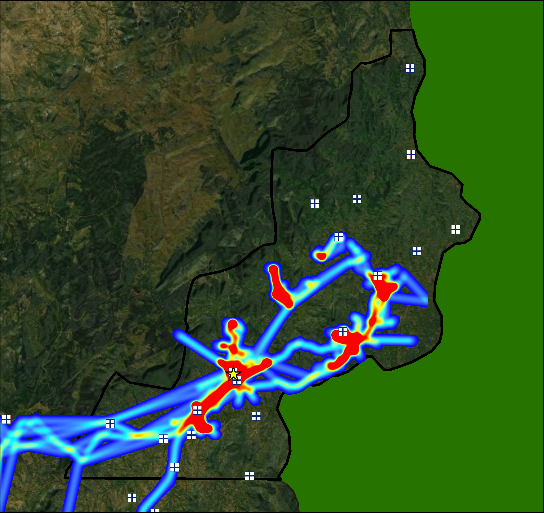

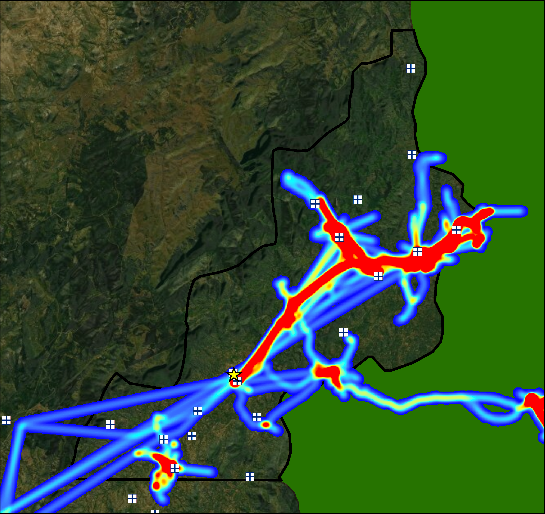

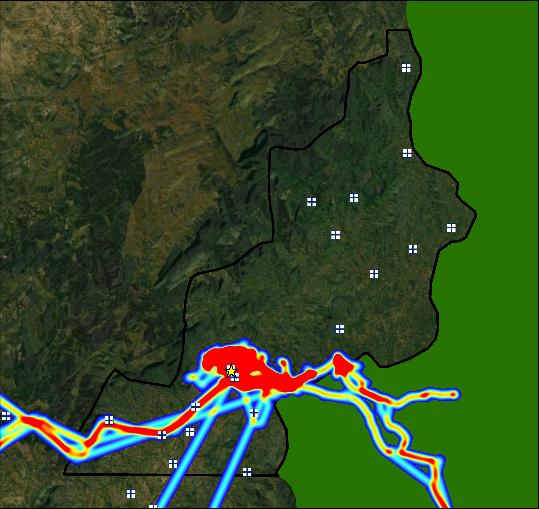

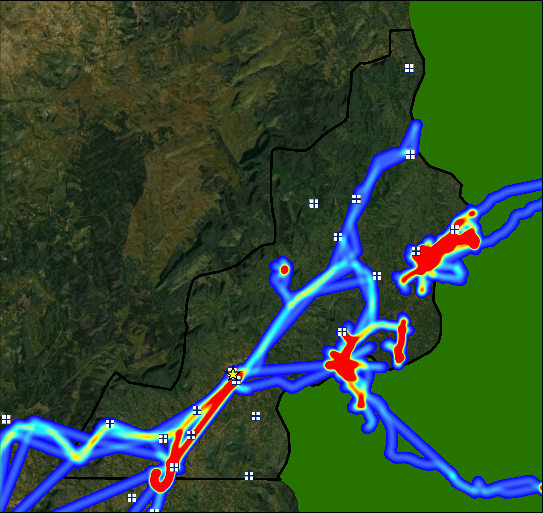

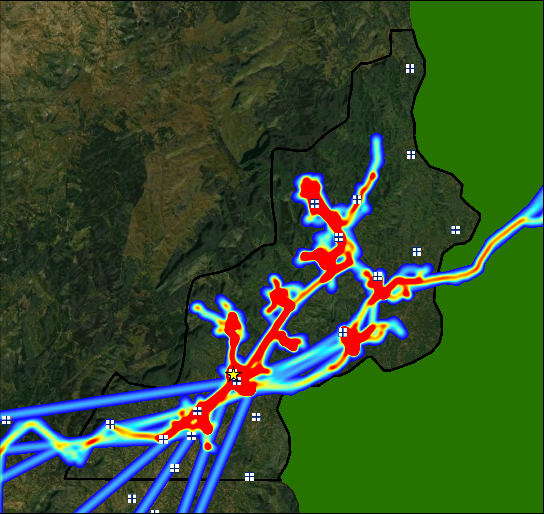

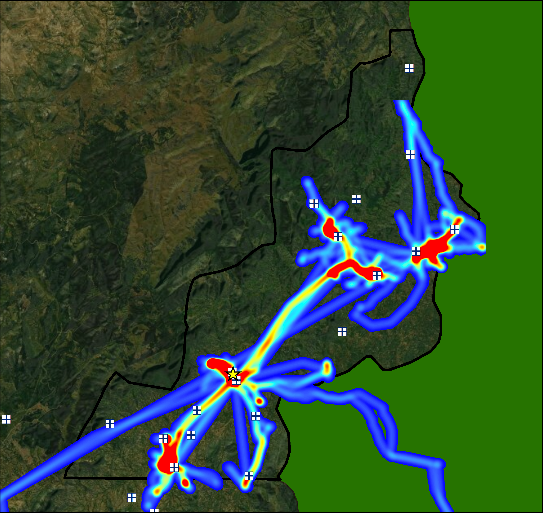

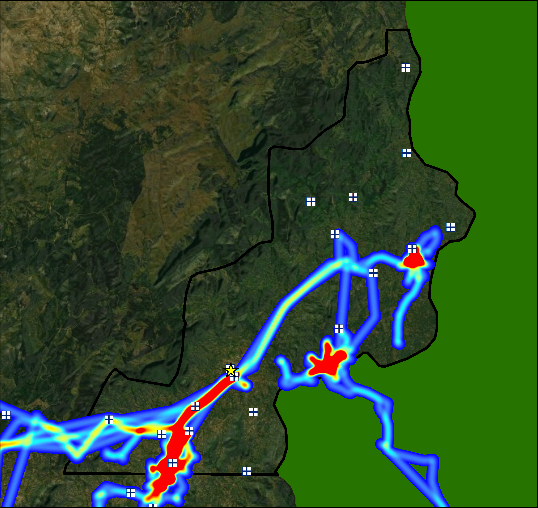

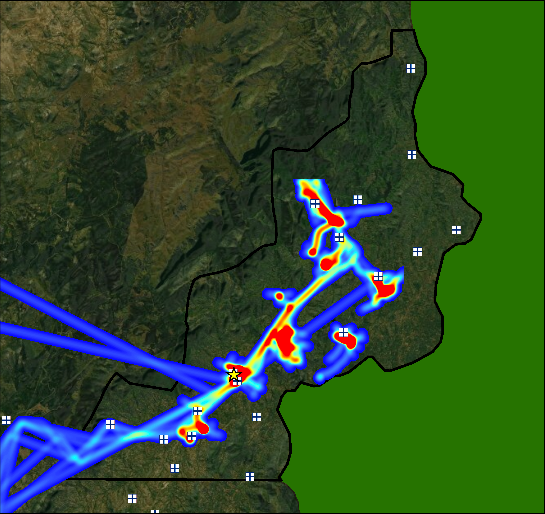

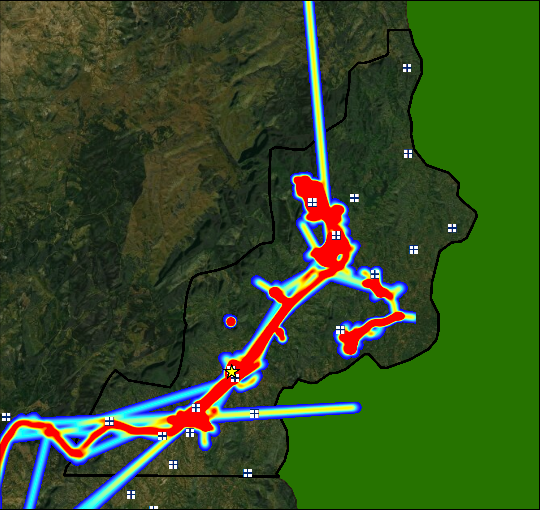

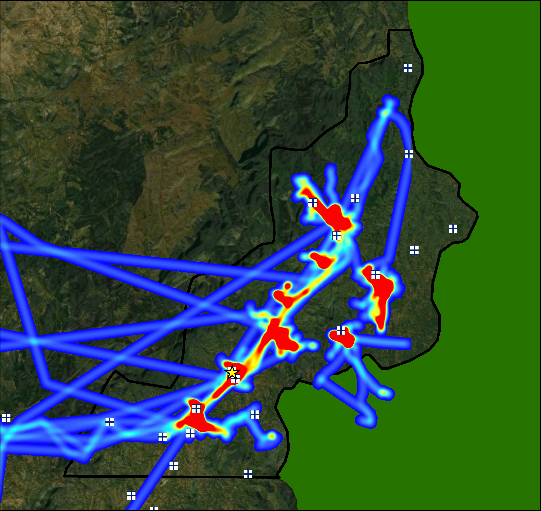

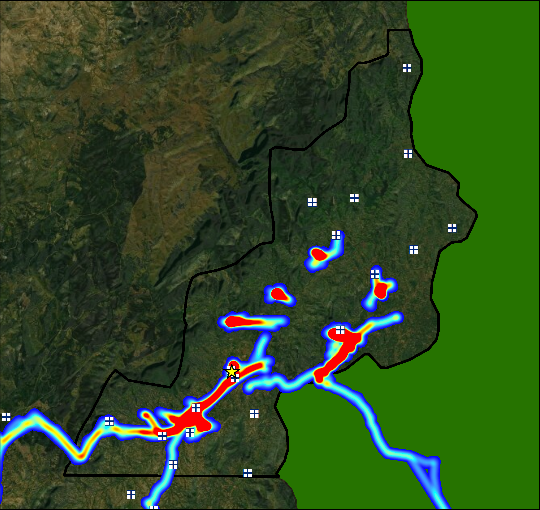


H

G

F

E

L

K

J

I

Figure S2: Intensity maps of population movement in Nchelenge District from June 2016 – May 2017 by cohort and season: A) longitudinal cohort, dry season [June, August, October], B) cross-sectional cohort, dry season [July, September, November], C) longitudinal cohort, rainy season [December, February, April], D) cross-sectional cohort, rainy season [January, March, May].


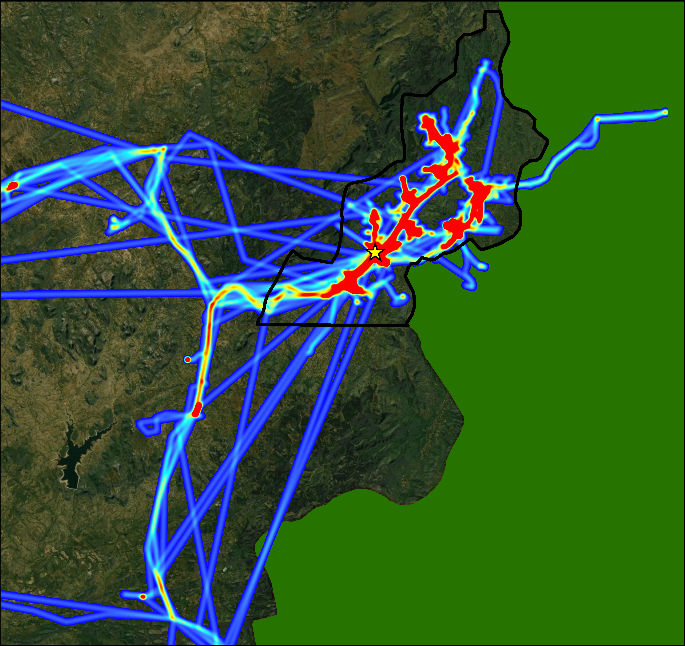

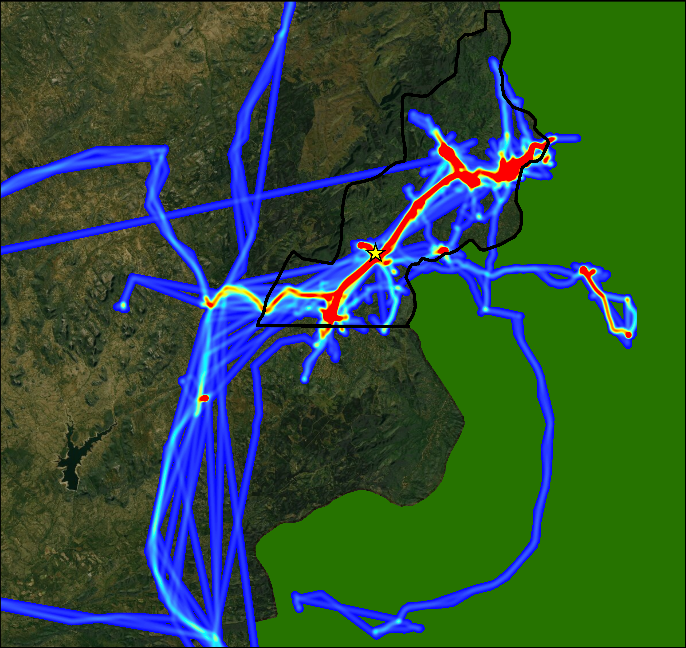

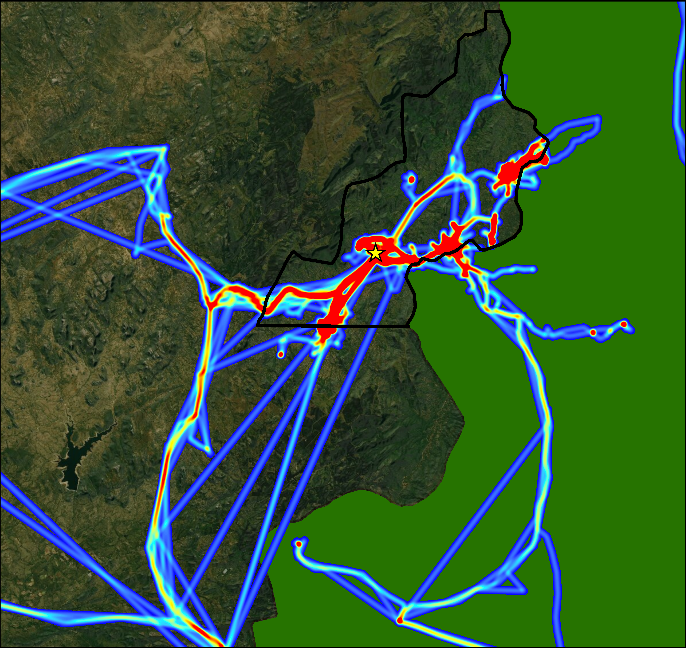

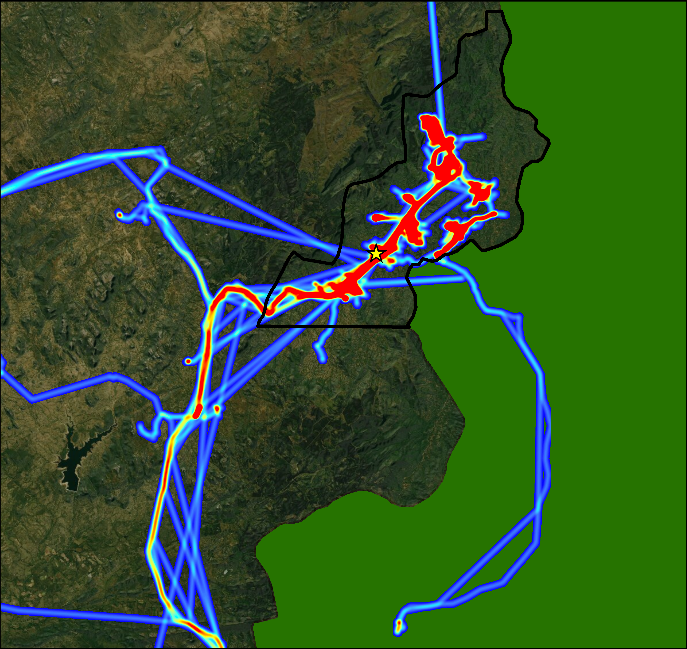


A

B

C

D
